# Features of asthma management: quantifying the patient perspective

**DOI:** 10.1186/1471-2466-7-16

**Published:** 2007-12-06

**Authors:** John Haughney, Monica Fletcher, Stephanie Wolfe, Julie Ratcliffe, Roger Brice, Martyn R Partridge

**Affiliations:** 1 University of Aberdeen, Department of General Practice and Primary Care Aberdeen, AB25 2AY, UK; 2 Education For Health, Chief Executive, Warwick, CV34 4AB, UK; 3 Thorpewood Medical Group, Respiratory Nurse, Norwich, NR7 9QL, UK; 4 University of Sheffield, Health Economics and Decision Science, Sheffield, S10 2TN, UK; 5 Adelphi Group LTD, Research Director, Macclesfield, SK10 5JB, UK; 6 Imperial College, Chair in Respiratory Medicine, London, W6 8RP, UK

## Abstract

**Background:**

In the management of asthma, features of care important to patients may not be fully appreciated. This study quantifies the importance of different features of asthma management from the patient perspective. This may assist in the development of personalised management strategies.

**Methods:**

We used the technique of discrete choice experiment (DCE). Patients over 18 years of age with asthma, prescribed and taking medicine at step 3 of the UK guidelines were recruited from 15 general (family) practices in three areas of the UK. 147 evaluable questionnaires were returned from a total of 348 sent out. The outcome measures were the relative importance to patients of features of asthma management and the impact of changes in asthma management, as measured by utility shift between the features tested.

**Results:**

The largest shift in mean utility values was recorded in "number of inhalers" and "use of inhaled steroid". Use of a personal asthma action plan was ranked next highest.

**Conclusion:**

This study suggests that adults with moderate or severe asthma would trade some improvements in symptom relief in favour of, for example, simpler treatment regimens that use as few inhalers as possible and a lower dose of inhaled steroid.

## Background

Patient "self management" or "self care," a concept that enables patients to take a guided but ultimately personal involvement in the management of their condition, is an increasingly debated element of healthcare provision. It is particularly relevant as the prevalence of long term conditions increases and growing numbers of people desire a more active role in their own care with a less paternalistic approach from healthcare professionals [1]. Effective self care has the potential to improve clinical outcomes and reduce use of healthcare resources [1,2].

Asthma is an ideal condition in which to strive for improved patient outcomes by optimising self management because it typically fluctuates over time, with symptoms and exacerbations that can potentially be minimised with self monitoring and appropriate adjustment of treatment [3,4]. Self management of asthma is currently suboptimal in many patients, with around 50% self managing in ways that differ from recommended guidance [5-7].

A key step in improving the self management of asthma is to understand what patients consider important. Patient education programmes designed to improve self care have traditionally centred on what health professionals consider to be important, for example, lung function, asthma symptoms and bronchodilator use in asthma [8]. Previous research has shown that patients have different perceptions of asthma compared to health professionals and that education tailored to meet patients' perceptions is more likely to change behaviour [7].

This study was designed to quantify the relative importance of features of the management of asthma from the patients' perspective. We used discrete choice experiment methodology, a type of conjoint analysis that has been shown to be a rigorous survey technique for eliciting preferences [9]. It is increasingly being used to identify patient and public preferences for health care [10,11]. The technique allows respondents to choose their preferred option between hypothetical scenarios designed to reflect the different attributes that real world decisions would contain, and to make trade offs between these attributes to reveal their preferences. This technique of revealing preference through choice is a truer representation of real life decision-making and as such may be a better tool for establishing preference than data based on the ranking or rating of individual components of asthma management [12].

A clearer understanding of such preferences may help healthcare professionals tailor an acceptable personalised management of asthma with their patient and consequently move nearer to controlled asthma [13].

A brief description of discrete choice experiment, a working example and a glossary of technical terms and jargon are provided at Additional file 1.

## Methods

We carried out a discrete choice experiment (DCE) to determine the characteristics of long term asthma management that patients consider most important, requiring them to make choices between hypothetical scenarios and thus reveal their preferences.

Ethical approval was granted by Warwickshire Local Research Ethics Committee on behalf of COREC UK reference number 04/Q2803/66.

### The study population and questionnaire

To ensure a reasonable spread both geographically and socio-economically, 15 general practices from three geographical areas of the United Kingdom (UK) (West of Scotland, Norfolk, Gloucestershire), with a total population of 116 000 patients, took part in the study. Nursing staff at each practice identified all patients on treatment step 3 or above in the British Asthma Guidelines (regular use of inhaled steroid and other therapies)[14] who had received a prescription for asthma in the last 12 months, were over 18 years of age, and were believed to be able to understand and complete the questionnaire used in the study. The patients identified were included in a practice held "asthma register". The diagnostic criteria for inclusion in this register were likely to be variable. In many cases, a diagnosis of asthma will have been given and accepted without formal, objective evidence of asthma. This scenario is consistent with standard UK practice. Patients on UK asthma guideline treatment step 3 or above were chosen because their asthma management, by definition, is more complex than those at treatment steps 1 and 2.

A sample was selected by allocating each patient a unique identification number and then by the use of a random number generator computer program. The number selected from each practice varied according to total eligible patient numbers, with a maximum of 30 patients per practice. A total of 348 questionnaires were mailed. A traditional power calculation is not appropriate in calculating a sample size for a DCE, where rules of thumb and experience drive the sample size decision. The accepted rule of thumb for our experimental design (nine tasks and two alternatives per task per respondent and no more than three levels in any one attribute) is that the sample size should be in excess of 83 [15].

**Figure 1:**
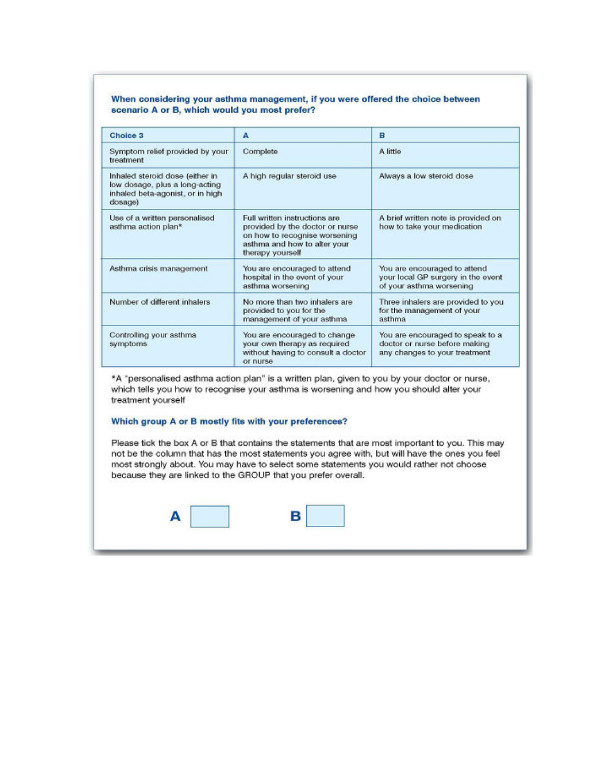
Example of question card.

The questionnaire presented respondents with nine pairs of choices (see Figure 1 and Additional file 2) – the discrete choice experiment. Socio-demographic information was also collected.

### Establishing the attributes and their levels for the discrete choice experiment

The key attributes for this discrete choice experiment were drawn from a previous study which included qualitative interviews with more than 400 patients with asthma [16]. We chose six attributes highlighted by patients as being the most important considerations in their long term asthma management. These were: importance of gaining relief of asthma symptoms from treatment; dose of inhaled steroid; the availability and content of a written personalised asthma action plan; locus of crisis (exacerbation) management; number of inhalers prescribed for routine use; and response to a deterioration.

We chose and assigned what we considered to be plausible and realistic levels for the six attributes that represent scenarios commonly found in asthma management. Table 1 lists the levels chosen for each of the attributes.

**Table 1:** Attributes and levels included in the study and constraints applied prior to analysis

*Attribute*	*Levels*	*Description*	*Constraints*
Symptom relief provided by your treatment	Completely Mostly A little	ORDINAL	Completely > Mostly > A little
Inhaled steroid dose	Always a low dose High dose when required but generally as little as possible High and regular steroid use	NOMINAL (with constraints)	Always low > Always high (no other assumptions made)
Use of a written personalised asthma action plan (PAAP)	Full written instructions are provided by your doctor or nurse on how to recognise worsening asthma and how to alter your therapy yourself Brief written note is provided on how to take your medication No written instructions are provided	NOMINAL	None
Asthma crisis management	You are encouraged to: Manage an asthma crisis yourself whenever possible Attend your local GP in the event of an asthma crisis Attend hospital in the event of an asthma crisis	NOMINAL (with constraints)	GP>Hospital Yourself>Hospital (no assumption on Yourself v GP)
Number of different inhalers	A single inhaler is provided to you which contains all the inhaled medication you need for the management of your asthma No more than two inhalers are provided for the management of your asthma Three inhalers are provided to you for the management of your asthma	ORDINAL	1>at most 2>3
Controlling your asthma symptoms	You are encouraged to: Change your own therapy in response to changes in your symptoms without consulting a doctor or a nurse Speak to a doctor or nurse before making changes to treatment	NOMINAL	None

A design program used in the statistical software SAS [17] was used. This software produces a manageable number of combinations of attributes and their respective levels (or scenarios) to develop a survey questionnaire, balancing the statistical requirements with the need to avoid overburdening the respondent with work. A total of nine pairs of choices were produced. For each pair of scenarios, respondents were asked to indicate the one they would most prefer when considering how their asthma should be managed (see Figure 1).

### Data analysis

Using the techniques described in Additional file 3, the overall relative importances of attributes at both individual and aggregate (group) levels, and shifts in utility values between each level within each attribute were calculated (see additional files).

## Results

A total of 148 questionnaires were returned after one reminder, of which one was returned blank, from a total of 348 sent out, giving a useable response rate of 43%. Table 2 summarises the sociodemographic characteristics of the study population, while Table 3 compares basic characteristics of responders with non-responders.

**Table 2:** Descriptive characteristics of respondents (n = 147)

***Characteristics***	***Mean (SD) or n (%)***
**Age ***(missing = 1)*	53.2 (16.2)
**Male gender**	48 (32.7)
**Asthma duration**	
< 12 months	0
1 to 4 years	14 (9.5)
5 to 10 years	33 (22.4)
More than 10 years	100 (68.0)
**English is first spoken language ***(missing = 2)*	143 (97.3)
**Difficulty of questionnaire ***(missing = 2)*	
Very	4 (2.7)
Moderately	20 (13.8)
Slightly	29 (20.0)
Not	92 (63.4)

**Table 3:** Characteristics of responders compared to non-responders

	**Responder**		**Non-responder**	
	
	***Number***	***Mean (SD) or percentage***	***Number***	***Mean (SD) or percentage***
Age	147	53.2 yrs (16.2)	201	45.2 yrs (15.9)
**Female Gender**	99	67%	129	64%

Non-responders, by definition, did not consent to their involvement in the study. Consequently, a more detailed comparison of characteristics or features of responders and non-responders was not possible.

The Relative Importance results are presented in Table 4.

**Table 4:** Relative Importance (RI) of Attribute Ranges Tested

**Attribute**	**Individual Level RI**	***Aggregate Level RI***
Number of Different Inhalers	**21.9%**	*29.3%*
Inhaled Steroid Dose	**20.3%**	*21.1%*
Use of a Written PAAP	**17.0%**	*12.3%*
Asthma Crisis Management	**15.2%**	*15.0%*
Controlling Your Asthma Symptoms	**14.4%**	*6.1%*
Symptom Relief Provided by Your Treatment	**11.3%**	*16.3%*

The outputs shown throughout are the means of the parameters calculated at the level of **individual **respondents. The means at **aggregate **level demonstrating relative importance were also calculated and are included for comparison, displaying where a non-homogenous response occurs. See Additional file 1.

**Figure 2:**
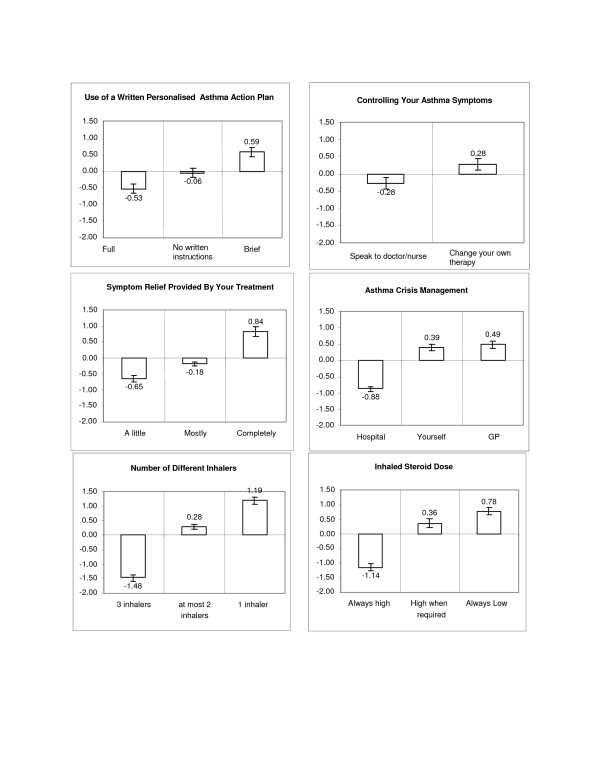
Mean utility values.

Figure 2 shows the importance that respondents placed on changes between different levels within-attributes. The **degree **of importance is seen by changes in utility values for levels within the attributes. All 11 successive within attribute transitions were statistically significant (P < 0.05), with the single exception of changing the management of an asthma crisis from 'yourself' to 'visiting a GP/nurse'. Those changes with the highest relative negative impact on respondents' views of their asthma management were:

• changing from 'no more than 2' to '3' inhalers,

• change in steroid dose from 'low but high when needed' to 'always high',

• being encouraged 'to visit a hospital for crisis management' rather than being encouraged 'to manage yourself' or 'attend the local GP surgery',

• symptom relief provided by current treatment changing from 'completely' to 'mostly',

• changing from 1 to 2 inhalers.

## Discussion

The study emphasises the importance of keeping treatment regimens simple. The results showed that adults with moderate or more severe asthma considered that a simple treatment regimen was the most important consideration in the long-term management of their condition, rather than symptom control without compromise. For example, two of the top five highest utility shifts between levels related to the number of inhalers they needed to use. Changing from 'no more than two' to 'three' inhalers had the highest relative negative impact on respondents' views of their asthma management. While noting the caveats of the relative importance analysis, number of inhalers was ranked the most important attribute of asthma management at both the aggregate (29.3%) and individual levels (21.9%), suggesting a reasonably homogenous view.

This preference for simpler treatment and fewer inhalers confirms in a more systematic and rigorous way preferences for "fewer drug treatments" and "just one inhaler" reported in a previous pan-European study [7] and confirms the findings from patient interviews in our previous study [16]. Asthma is only one part of people's lives and treatments that may need to be taken for decades should be offered in the simplest format. Willingness to pay from the patients' perspective – another factor that may influence treatment preference – was not addressed in this study; the cost of therapy to patients may be less important in the UK than in other healthcare settings; it was not rated highly as an issue in our qualitative study [16].

The factor that patients rated as being of next highest relative importance, and which had the second greatest utility shift, was the dose of inhaled steroid. Scope for lowering the steroid dose without loss of asthma control has previously been described [18] and the addition of an inhaled long-acting beta agonist often permits better control and use of a lower dose of inhaled steroid [14].

Use of a personalised asthma action plan came next in patients' ranking of relative importance of the attributes of asthma management that they were asked about. A discouragingly small number, only 12 (8%) of respondents, indicated that they held a written personalised asthma action plan – two centres each accounted for three of these patients and a further six practices each had one patient with a plan. This low number of patients with an action plan is similar to that found in previous studies [19] and is disappointing, especially because it has previously been shown that even those without plans would feel comfortable adjusting therapy themselves [16]. Written asthma action plans have been shown both to improve outcomes [3] and to improve compliance with asthma therapy [20], to be cost-effective [21] and are strongly recommended in asthma guidelines [14]. It may be that lack of familiarity with the nature and benefits of using a personalised plan, by both medical professionals and patients, may have influenced these results and that a greater knowledge would increase the popularity and use of what may be the single most important non-therapeutic intervention in asthma management. In this study, patients indicate a desire for "brief" rather than "full" written instructions.

The next ranked factor was asthma crisis management. The utility analysis showed that patients preferred to avoid attending hospital even in the event of a crisis, a theme we have reported in a different disease area and population [22]. Knowledge of patient preference can inform the clinician but will not, of course, be the only factor to consider when deciding how and where to manage an acute exacerbation of asthma.

Perhaps surprisingly, controlling asthma symptoms was ranked lower in patients' ranking of importance, and relief of symptoms was considered least important in the range of attributes tested. However, this does not mean that people with asthma do not consider symptom relief important, but indicates that respondents considered it less important than the other attributes of asthma management they were asked to rank. This suggests that patients were prepared, at least to some extent, to trade off elements of efficacy for what they perceived to be other benefits, such as lower doses of inhaled steroids.

Both 'asthma crisis management' and 'controlling your asthma symptoms' had higher relative importance statistics when determined by the individual level method than by the aggregate level method. This means that there was a division of opinion within respondents as to which level in each of these two attributes was the most desirable.

There was some variation between respondents in the extent to which they wanted to manage their own asthma symptoms. Nearly two-thirds put a higher utility value on being encouraged to 'change your own therapy' than 'speak to a doctor or nurse before making changes to treatment' in the attribute of controlling asthma symptoms. This indicates a split between patients wanting a collaborative/active role in making changes to their asthma therapy and those wanting a more passive role, at a similar level to that reported previously [23].

One of the greatest strengths of this study is the use of discrete choice experiment methodology, which is a rigorous method of eliciting preferences. Previous studies have demonstrated that respondents tend to behave in an internally valid and consistent manner when answering DCE questions [24]. The study explored patients' preferences between only the attributes and levels that were offered, but these had been identified as being important from patients interviewed in a previous study [16]. The majority of the respondents found the questionnaire easy to complete, although it is possible that the type of questionnaire and the task, which is likely to have been unfamiliar to recipients, influenced the overall response rate.

Another possible limitation to the study is that the majority of respondents were female (65%) and aged over 55 years (48%). However, this is similar to previous studies exploring adult asthma patients' attitudes to their treatment [9,23]. Responders were generally older (mean age 55 years) than non-responders (mean age 45 years) (P < 0.01), but there was no statistically significant difference in gender between respondents and non-respondents (P = 0.3%).

## Conclusion

Taking a flexible, patient-centred approach to asthma management means focusing on issues that patients consider important. Our study indicates that this means making treatment as simple as possible, with as few medications and inhalers as can achieve symptom control – ideally fewer than three, or even two, inhalers. It also means using the lowest dose of inhaled steroid that can effectively control asthma and avoiding hospitals for emergency care, as well as minimising asthma symptoms. There is clearly room for improvement in increasing the number of patients receiving personalised asthma management plans, which should improve outcomes by increasing compliance.

## Competing interests

John Haughney has received fees from AstraZeneca, Boehringer Ingelheim and Merck Sharp & Dohme for speaking at meetings and from AstraZeneca, GlaxoSmithKline, Merck Sharp & Dohme, Novartis and Schering Plough for consulting. Monica Fletcher has received reimbursement from AstraZeneca, Schering Plough, Altana and Novartis for attending international conferences, fees for speaking at meetings and funds for research. Stephanie Wolfe has received fees from Merck Sharp & Dohme for staff training, Novartis for consulting and AstraZeneca, GlaxoSmithKline and Merck Sharp & Dohme for speaking at meetings. Julie Ratcliffe has no competing interests. Roger Brice has no competing interests. Martyn Partridge has received fees for lecturing from AstraZeneca, GlaxoSmithKline, Novartis and Vitaris. He has in addition received sponsorship to attend European Respiratory Society and American Thoracic Society meetings from AstraZeneca and Novartis, and the Astra Foundation have supported a non-clinical Research Fellow.

## Authors' contributions

JH, MP and MF conceived and designed the study, interpreted the data and wrote and reviewed the manuscript. SW supervised the processes of the study, interpreted the data and reviewed the manuscript. JR helped conceive and design the study, interpreted the data and reviewed the manuscript. RB carried out the analyses, interpreted the data and reviewed the manuscript. All authors read and approved the final manuscript.

## Pre-publication history

The pre-publication history for this paper can be accessed here:



## Supplementary Material

Additional file 1 **Discrete choice experiment in practice: a lay example**. Discrete choice experiment, a form of conjoint analysis, allows not only a rank order of importance to be identified but also allows the distance between features, the degree of importance, to be measured. This very example was used in our study to introduce participants to the concept. Click here for file

Additional file 2 **More examples of SET CHOICES given to patients**. The questionnaire presented respondents with nine pairs of choices relating to their asthma Click here for file

Additional file 3 **Analysis of the direct choice experiment**. The overall relative importances of attributes at both individual and aggregate (group) levels, and shifts in utility values between each level within each attribute were calculated Click here for file
