# Supplementary material for: Features of asthma management: quantifying the patient perspective
Source: BMC Pulm Med. 2007 Dec 6;7:16. doi: 10.1186/1471-2466-7-16 (PMC2231386; doi:10.1186/1471-2466-7-16)
Supplement: Additional file 1 — Discrete choice experiment in practice: a lay example. Discrete choice experiment, a form of conjoint analysis, allows not only a rank order of importance to be identified but also allows the distance between features, the degree of importance, to be measured. This very example was used in our study to introduce participants to the concept. [file 1471-2466-7-16-S1.doc]

**Additional file 1**

**Discrete choice experiment in practice: a lay example**

Consider you are going on holiday and you need to book a flight. You may wish to consider certain features of your holiday, such as your departure airport and the length of your flight, the food, hotel and weather and, of course, the cost. In an ideal world, many would ideally want a short flight from a convenient airport with pleasant weather in a comfortable hotel with agreeable food at modest cost. But in reality, choices and compromises have to be made. What is really important? And what are you willing to trade to achieve the most desirable features? Discrete choice experiment, a form of conjoint analysis, allows not only a **rank order** of **importance** to be identified but also allows the **distance** between features, the degree of importance, to be measured. This very example was used in our study to introduce participants to the concept. See below.

Practice question: Planning a holiday

| **Choice 1** | **A** | **B** |
| --- | --- | --- |
| *Length of flight* | I prefer a short flight | I don’t really mind how long the flight takes |
| *Weather* | My destination must be hot and sunny | As long as its better than home, I don’t really bother about the weather |
| *Hotel* | I always prefer a luxury hotel | I’m happy in a simple hotel |
| *Cost* | The price is important to me | I’m happy to pay for extra quality |
| *Flight* | I prefer to fly from my local airport | I’m willing to travel to get a cheaper flight |
| *Food* | I’ll eat anything | I like to have the food I’m used to on holiday |

Which group A or B mostly fits with your preferences?

Please tick the box A or B that contains the statements that are most important to you. This may not be the column that has the most statements you agree with, but will have the ones you feel most strongly about. You may have to select some statements you would rather not choose because they are linked to the GROUP that you prefer overall

A B

|  |  |
| --- | --- |

**Aggregate and individual responses**

Let us presume we interview 100 people about their favourite pet. In our survey, 50 strongly prefer cats and 50 strongly prefer dogs. If we present **aggregate** level data, then no preference is clear: dogs are as popular cats, the group has no preference. This result would also be achieved if all our respondents stated they liked cats and dogs equally. If the data is presented at individual level, then it is clear that individuals hold strong, but not homogenous, preferences for either cats or dogs.

**Glossary of terms**

Attribute: A feature tested. For example, the number of inhalers.

Level: A grading within an attribute eg 1, 2 or 3. This grading is said to be ordinal (2 is >1 and < 3). Levels may, of course, be nominal when there is no logical progression. For example, which colour do you prefer? Red, blue or green?

Relative importance: a means of ranking the attributes tested

Utility shift: a numerical value allowing comparison between changes in levels tested. The greater the utility shift, the greater the importance of the shift from one level to another. Note that utility values are unique to each DCE and cannot be used for comparison with features in other DCEs
