# Supplementary material for: Features of asthma management: quantifying the patient perspective
Source: BMC Pulm Med. 2007 Dec 6;7:16. doi: 10.1186/1471-2466-7-16 (PMC2231386; doi:10.1186/1471-2466-7-16)
Supplement: Additional file 2 — More examples of SET CHOICES given to patients. The questionnaire presented respondents with nine pairs of choices relating to their asthma [file 1471-2466-7-16-S2.doc]

**Additional file 2 more examples of SET CHOICES given to patients**

When considering your asthma management, if you were offered the choice between scenario A or B, which would you most prefer?

| **Choice 1** | **A** | **B** |
| --- | --- | --- |
| *Relief of asthma symptoms provided by treatment* | Mostly | A little |
| *Regular use an of inhaled steroid (either in low dosage, plus a long acting inhaled beta agonist), or in high dosage* | A high regular steroid use | A high steroid use when required but generally as little as possible |
| *Use of a written personalised asthma action plan[[1]](#footnote-2)** | A brief written note is provided on how to take your medication | Comprehensive written instructions are provided by the doctor or nurse on how to recognise worsening asthma and how to alter your therapy yourself |
| *Asthma management* | You are encouraged to attend hospital in the event of your asthma worsening | You are encouraged to attend your local GP surgery in the event of your asthma worsening |
| *Number of different inhalers* | Three inhalers are provided to you for the management of your asthma | A single inhaler is provided to you which contains all the inhaled medication you need for the management of your asthma[[2]](#footnote-3)* |
| *Controlling your asthma symptoms* | You are encouraged to speak to a doctor or nurse before making any changes to your treatment | You are encouraged to change your own therapy as required without having to consult a doctor or nurse |

Which group A or B mostly fits with your preferences?

Please tick the box A or B that contains the statements that are most important to you. This may not be the column that has the most statements you agree with, but will have the ones you feel most strongly about. You may have to select some statements you would rather not choose because they are linked to the GROUP that you prefer overall

A B

|  |  |
| --- | --- |

When considering your asthma management, if you were offered the choice between scenario A or B, which would you most prefer?

| **Choice 2** | **A** | **B** |
| --- | --- | --- |
| *Relief of asthma symptoms provided by treatment* | Completely | Mostly |
| *Regular use an of inhaled steroid (either in low dosage, plus a long acting inhaled beta agonist), or in high dosage* | Always a low steroid dose | A high steroid use when required but generally as little as possible |
| *Use of a written personalised asthma action plan.[[3]](#footnote-4)** | No written instructions are provided | Comprehensive written instructions are provided by the doctor or nurse on how to recognise worsening asthma and how to alter your therapy yourself |
| *Asthma management* | You are encouraged to manage a worsening of your asthma wherever possible | You are encouraged to manage a worsening of your asthma wherever possible |
| *Number of different inhalers* | Three inhalers are provided to you for the management of your asthma | A single inhaler is provided to you which contains all the inhaled medication you need for the management of your asthma[[4]](#footnote-5)* |
| *Controlling your asthma symptoms* | You are encouraged to change your own therapy as required without having to consult a doctor or nurse | You are encouraged to change your own therapy as required without having to consult a doctor or nurse |

Which group A or B mostly fits with your preferences?

Please tick the box A or B that contains the statements that are most important to you. This may not be the column that has the most statements you agree with, but will have the ones you feel most strongly about. You may have to select some statements you would rather not choose because they are linked to the GROUP that you prefer overall

A B

|  |  |
| --- | --- |

When considering your asthma management, if you were offered the choice between scenario A or B, which would you most prefer?

| **Choice 4** | **A** | **B** |
| --- | --- | --- |
| *Relief of asthma symptoms provided by treatment* | Mostly | A little |
| *Regular use an of inhaled steroid (either in low dosage, plus a long acting inhaled beta agonist), or in high dosage* | A high steroid use when required but generally as little as possible | A high steroid use when required but generally as little as possible |
| *Use of a written personalised asthma action plan.[[5]](#footnote-6)** | No written instructions are provided | Comprehensive written instructions are provided by the doctor or nurse on how to recognise worsening asthma and how to alter your therapy yourself |
| *Asthma management* | You are encouraged to attend your local GP surgery in the event of your asthma worsening | You are encouraged to manage a worsening of your asthma wherever possible |
| *Number of different inhalers* | No more than two inhalers are provided to you for the management of your asthma | No more than two inhalers are provided to you for the management of your asthma |
| *Controlling your asthma symptoms* | You are encouraged to change your own therapy as required without having to consult a doctor or nurse | You are encouraged to change your own therapy as required without having to consult a doctor or nurse |

Which group A or B mostly fits with your preferences?

Please tick the box A or B that contains the statements that are most important to you. This may not be the column that has the most statements you agree with, but will have the ones you feel most strongly about. You may have to select some statements you would rather not choose because they are linked to the GROUP that you prefer overall

A B

|  |  |
| --- | --- |

1. ^*^ A “personalised asthma action plan” is a written plan, given to you by your doctor or nurse, which tells you how to recognise your asthma is worsening and how you should alter your treatment yourself [↑](#footnote-ref-2)
2. ^*^A “single inhaler” is an inhaler, which contains all the different inhaled medications you require in just one inhaler [↑](#footnote-ref-3)
3. ^*^ A “personalised asthma action plan” is a written plan, given to you by your doctor or nurse, which tells you how to recognise your asthma is worsening and how you should alter your treatment yourself [↑](#footnote-ref-4)
4. ^*^ A “single inhaler” is an inhaler, which contains all the different inhaled medications you require in just one inhaler [↑](#footnote-ref-5)
5. ^*^ A “personalised asthma action plan” is a written plan, given to you by your doctor or nurse, which tells you how to recognise your asthma is worsening and how you should alter your treatment yourself

   ^*^ A “single inhaler” is an inhaler, which contains all the different inhaled medications you require in just one inhaler [↑](#footnote-ref-6)
