# Supplementary material for: Features of asthma management: quantifying the patient perspective
Source: BMC Pulm Med. 2007 Dec 6;7:16. doi: 10.1186/1471-2466-7-16 (PMC2231386; doi:10.1186/1471-2466-7-16)
Supplement: Additional file 3 — Analysis of the direct choice experiment. The overall relative importances of attributes at both individual and aggregate (group) levels, and shifts in utility values between each level within each attribute were calculated [file 1471-2466-7-16-S3.doc]

**Additional file 3**

**Analysis of the direct choice experiment**

A multinomial logit model with dummy variables was specified with the choice responses as the binary dependant variable and the differences in levels for each attribute within each choice as the independent variables. For each individual respondent, the function to be estimated was of the following form:

p_k_ = exp(x_k_’ β_I_) / Σ_jexp_(x_j_ β_I_)

where:
p_k_ = the probability of an individual choosing the kth scenario in a particular choice task x_j_ = a vector of values describing the jth alternative in that choice task
β_I_ = a vector of utility values for the ith respondent. These utility values are assumed to have the multivariate normal distribution,

β_I_ ~ Normal (α, D)
where:
α = a vector of means of the distribution of individuals’ utility values
D = a matrix of variances and covariances of the distribution of utility values across individuals

The data were analysed using the CBC/HB analysis module from Sawtooth Software. (http://www.sawtoothsoftware.com/home.shtml). This uses an iterative Monte Carlo Markov chain iterative procedure with a Metropolis Hastings algorithm [1].

**Calculating the relative importance of variation for each individual**

The relative importance of variation across the range of each of nine attributes was calculated for each individual respondent. This was calculated for each individual respondent using the following formula :

RI_ij_ (%) = 100 x (U_ijmax_ – U_ijmin_) / Σ (U_ijmax_ – U_ijmin_)

Where U_ijmax_ = maximum utility value of levels within the i^th^ attribute and U_imin_ = minimum utility value of levels within the i^th^ attribute for the ith respondent.

The overall relative importance of attributes was calculated at both the individual and aggregate sample levels. The former is defined as ΣRI_ij_/n (i.e. the mean of the importances of each attribute for each respondent) and the latter by applying the ‘importance’ formula (above) to the means of the utility values across the whole sample (i.e. treating the means for utility values across all individuals as though they were from one individual). A difference between attribute importances, as measured by these two methods, is an indication of the extent of heterogeneity within the respondent sample.

**Appendix references**

1. Chib S, Greenberg E: **Understanding the Metropolis–Hastings algorithm.** *Am Stat* 1995,**49**(4):327–335.
